# 原发免疫性血小板减少症患者血栓风险预测模型的构建与评价

**DOI:** 10.3760/cma.j.cn121090-20250507-00213

**Published:** 2026-02

**Authors:** 佳慧 毛, 琳 王, 艳 石, 琳琳 邵, 宇 侯, 明 侯

**Affiliations:** 山东大学齐鲁医院血液科，济南 250012 Department of Hematology, Qilu Hospital of Shandong University, Jinan 250012, China

**Keywords:** 紫癜，血小板减少性，特发性, 血栓形成, 危险因素, 预测模型, Purpura, thrombocytopenic, idiopathic, Thrombosis, Risk factors, Prediction model

## Abstract

**目的:**

分析原发免疫性血小板减少症（ITP）患者发生血栓的危险因素，在此基础上构建血栓预测模型并对模型进行评价。

**方法:**

回顾性分析2018年1月至2022年12月于山东大学齐鲁医院住院治疗的334例ITP患者的临床资料，通过Logistic回归分析发生血栓的独立危险因素并构建预测模型。

**结果:**

334例ITP患者中40例（12.0％）发生血栓，其中男18例，女22例。动脉血栓、静脉血栓、混合血栓发生率分别为9.58％（32/334）、1.80％（6/334）和0.60％（2/334）。单因素分析显示，年龄、ITP病程>1年、合并高血压、合并冠心病、合并糖尿病、PLT均是ITP患者发生血栓的危险因素（*P*值均<0.05）。多因素分析显示，年龄、ITP病程>1年、合并冠心病、PLT均是发生血栓的独立危险因素（*P*值均<0.05）。列线图预测模型的受试者操作特征曲线的曲线下面积为0.80（95％*CI*：0.72～0.88），校准曲线显示列线图的预测与实际血栓发生率有良好的一致性，Hosmer-Lemeshow拟合优度检验显示*χ*^2^＝5.838，*P*＝0.665。

**结论:**

本研究构建了ITP患者发生血栓的列线图预测模型，有助于识别易发生血栓的高危患者。

## Abstract (en)

**Objective:**

To analyze the risk factors for thrombosis in patients with primary immune thrombocytopenia（ITP）, construct a prediction model for thrombosis, and evaluate its performance.

**Methods:**

The clinical data of 334 ITP patients hospitalized at Qilu Hospital of Shandong University from January 2018 to December 2022 were retrospectively analyzed. Logistic regression was used to identify independent risk factors for thrombosis and construct the prediction model.

**Results:**

Among 334 ITP patients, 40（12.0％）developed thrombosis, including 18 males and 22 females. The incidence rates of arterial thrombosis, venous thrombosis, and mixed thrombosis were 9.58％（32/334）, 1.80％（6/334）, and 0.60％（2/334）, respectively. Univariate analysis showed that age, ITP duration >1 year, comorbid hypertension, coronary heart disease, diabetes, and PLT were risk factors for thrombosis in ITP patients（all *P*<0.05）. Multivariate analysis revealed that age, ITP duration >1 year, comorbid coronary heart disease, and PLT were independent risk factors for thrombosis（all *P*<0.05）. The area under the ROC curve for the nomogram prediction model was 0.80（95％ *CI*: 0.72–0.88）, and the calibration curve showed good consistency between the predicted and actual thrombosis rates in ITP patients. The Hosmer-Lemeshow goodness-of-fit test showed *χ*^2^＝5.838, *P*＝0.665.

**Conclusion:**

This study developed a nomogram prediction model for thrombosis in ITP patients, which can help identify high-risk patients.

原发免疫性血小板减少症（ITP）是临床最常见的获得性自身免疫性出血性疾病，以外周血PLT降低为主要特点[1]。近年来国内外研究发现，ITP患者动、静脉血栓发生率均高于普通人群[2]–[4]。为深入探索ITP患者发生血栓的危险因素，本研究纳入2018年1月至2022年12月于山东大学齐鲁医院住院治疗的334例ITP患者，回顾性分析ITP患者发生血栓的危险因素，构建风险预测模型并对模型进行相应评价，以便及时筛查高风险人群并对ITP患者发生血栓的危险因素尽早干预，降低血栓发生风险。

## 病例与方法

1. 病例：本研究回顾性分析2018年1月至2022年12月于山东大学齐鲁医院住院治疗的334例ITP患者，诊断标准依据《成人原发免疫性血小板减少症诊断与治疗中国指南（2020年版）》[5]，具体为：至少连续2次血常规检查示PLT减少，外周血涂片镜检血细胞形态无明显异常；脾脏一般不增大；骨髓检查：细胞形态学特点为巨核细胞增多或正常，伴成熟障碍；须排除其他继发性血小板减少症。纳入的患者住院治疗期间主要临床资料完整。334例ITP患者按照是否发生血栓分为有血栓组和无血栓组。

2. 临床资料：包括患者的一般资料（性别、年龄、身高、体重、病程、是否感染、是否卧床超过3 d）、既往史、个人史、实验室检查（WBC、HGB、PLT、甘油三酯、低密度脂蛋白胆固醇、高密度脂蛋白胆固醇、总胆固醇）、治疗情况［一线治疗包括糖皮质激素（地塞米松、泼尼松）和静脉注射免疫球蛋白（IVIg），二线治疗包括促血小板生成药物（重组人血小板生成素、艾曲泊帕、阿伐曲泊帕、罗米司亭、海曲泊帕）、利妥昔单抗、脾切除术，其他药物：达那唑、咖啡酸］。

3. 观察指标：详细记录患者的血栓事件，动脉血栓事件包括急性心肌梗死、脑栓塞、短暂性脑缺血发作（TIA）、外周动脉血栓，静脉血栓事件包括肺栓塞（PE）、深静脉血栓（DVT）、浅静脉血栓、肌间静脉血栓、颅内静脉窦血栓、门静脉血栓（PVT）、肠系膜静脉血栓。混合型血栓即动脉血栓和静脉血栓同时发生。急性心肌梗死的诊断参照《急性ST段抬高型心肌梗死诊断和治疗指南（2019）》[6]，缺血性脑卒中的诊断参照《中国急性缺血性脑卒中诊治指南2018》[7]，肺栓塞的诊断参照《急性肺栓塞多学科团队救治中国专家共识》[8]，深静脉血栓常发生于下肢，因此需结合下肢静脉超声判断。B超、CT/CT血管造影（CTA）、磁共振成像（MRI）/磁共振血管成像（MRA）/磁共振静脉成像（MRV）等影像学检查有助于确定血栓事件并对其进行定位。

4. 随访：采用查阅门诊、住院电子病历及电话进行随访，随访的截止日期为2023年12月31日，中位随访28（5～60）个月。

5. 统计学处理：采用SPSS 26.0软件进行数据处理和分析，采用Shapiro-Wilk检验判断计量资料是否符合正态分布，符合正态分布的数据以*x*±*s*表示，采用*t*检验进行组间比较；不符合正态分布的数据以*M*（*Q*_1_，*Q*_3_）或*M*（范围）表示，采用Mann-Whitney *U*检验进行组间比较。计数资料用例数（％）表示，组间比较采用卡方检验或Fisher精确检验。采用单因素Logistic回归分析，差异有统计学意义（*P*<0.05）的变量即为ITP患者发生血栓的危险因素。将单因素分析结果中*P*<0.05的变量纳入多因素Logistic回归分析，差异有统计学意义（*P*<0.05）的变量即为ITP患者发生血栓的独立危险因素。将独立危险因素作为预测因子纳入风险预测模型，使用R 4.2软件构建列线图预测模型，并对模型进行评价。

## 结果

1. 临床资料：本研究共纳入334例ITP患者，男119例（35.6％），女215例（64.4％）。新诊断ITP 130例（38.9％），持续性ITP 66例（19.8％），慢性ITP 138例（41.3％）。血栓组40例（12.0％），无血栓组294例（88.0％），血栓组与无血栓组的一般资料比较显示，年龄、ITP病程>1年、合并高血压、合并冠心病、合并糖尿病、PLT的差异均有统计学意义（*P*值均<0.05）。两组患者性别、体重指数、合并感染、吸烟史、饮酒史、WBC、HGB、高密度脂蛋白胆固醇、低密度脂蛋白胆固醇、甘油三酯、总胆固醇的差异均无统计学意义（*P*值均>0.05）（表1）。既往治疗方面，两组患者接受糖皮质激素、IVIg、促血小板生成药物、利妥昔单抗、脾切除术、达那唑、咖啡酸治疗的差异均无统计学意义（*P*值均>0.05）（表1）。

**表1:** 血栓组、无血栓组原发免疫性血小板减少症（ITP）患者的临床特征比较

临床特征	血栓组（40例）	无血栓组（294例）	统计量	*P*值
男性［例（％）］	18（45.0）	101（34.4）	1.740（*χ*^2^值）	0.187
年龄［*M*（*Q*_1_，*Q*_3_）］	63（54，71）	51（32，61）	−4.675（*Z*值）	<0.001
体重指数［*M*（*Q*_1_，*Q*_3_）］	25.3（22.3，27.1）	24.6（21.8，27.2）	−0.670（*Z*值）	0.503
ITP病程>1年［例（％）］	27（67.5）	110（37.4）	13.172（*χ*^2^值）	<0.001
有吸烟史［例（％）］	6（15.0）	33（11.2）	0.189（*χ*^2^值）	0.663
有饮酒史［例（％）］	4（10.0）	16（5.4）	0.616（*χ*^2^值）	0.433
合并疾病［例（％）］				
高血压	17（42.5）	58（19.7）	10.486（*χ*^2^值）	0.001
冠心病	16（40.0）	16（5.4）	44.632（*χ*^2^值）	<0.001
糖尿病	12（30.0）	31（10.5）	11.882（*χ*^2^值）	0.001
感染	7（17.5）	48（16.3）	0.035（*χ*^2^值）	0.851
卧床超过3 d［例（％）］	3（7.5）	38（12.9）	0.524（*χ*^2^值）	0.469
实验室检查［*M*（*Q*_1_，*Q*_3_）］				
PLT（×10^9^/L）	41（18，144）	8（3，23）	−5.235（*Z*值）	<0.001
WBC（×10^9^/L）	6.55（4.97，9.47）	6.21（4.63，8.77）	−0.649（*Z*值）	0.516
HGB（g/L）	130（118，136）	124（105，138）	−1.134（*Z*值）	0.257
LDL-C（mmol/L）	2.40（1.75，3.21）	2.60（2.06，3.30）	−1.313（*Z*值）	0.189
HDL-C（mmol/L）	1.06（0.85，1.29）	1.11（0.92，1.34）	−1.356（*Z*值）	0.175
总胆固醇（mmol/L）	4.26（3.33，4.93）	4.50（3.75，5.35）	−1.356（Z值）	0.175
甘油三酯（mmol/L）	1.39（1.09，2.01）	1.38（0.95，1.92）	−0.660（*Z*值）	0.509
既往治疗情况				
糖皮质激素［例（％）］	25（62.5）	186（63.3）	0.009（*χ*^2^值）	0.925
IVIg［例（％）］	7（17.5）	66（22.4）	0.505（*χ*^2^值）	0.477
促血小板生成药物［例（％）］	12（30.0）	90（30.6）	0.006（*χ*^2^值）	0.937
利妥昔单抗［例（％）］	6（15.0）	32（10.9）	0.254（*χ*^2^值）	0.614
脾切除术［例（％）］	2（5.0）	8（2.7）	0.089（*χ*^2^值）	0.765
达那唑［例（％）］	4（10.0）	30（10.2）	<0.001（*χ*^2^值）	1.000
咖啡酸［例（％）］	3（7.5）	44（15.0）	1.623（*χ*^2^值）	0.203

**注** PLT：血小板计数；WBC：白细胞计数；HGB：血红蛋白；LDL-C：低密度脂蛋白胆固醇；HDL-C：高密度脂蛋白胆固醇；IVIg：静脉注射免疫球蛋白

2. 血栓发生情况及部位：40例患者发生血栓，共发生44例次血栓事件（包括动脉血栓35例次，静脉血栓9例次）。动脉血栓包括脑梗死29例次（65.9％），急性心肌梗死5例（11.4％），肺动脉栓塞1例次（2.3％）。静脉血栓包括颅内静脉窦血栓3例次（6.8％），下肢肌间静脉血栓5例次（11.4％），大隐静脉血栓1例次（2.3％）。2例患者合并混合血栓，其中1例患者合并脑梗死、肺动脉栓塞、下肢肌间静脉血栓和大隐静脉血栓，另1例合并脑梗死和下肢肌间静脉血栓。动脉血栓发生率9.58％（32/334），静脉血栓发生率1.80％（6/334），混合血栓发生率0.60％（2/334）。分别有15％（18/119）的男性患者和10％（22/215）的女性患者发生血栓。发生血栓时，14例患者PLT≤30×10^9^/L，10例患者PLT为（31～50）×10^9^/L，10例患者PLT为（51～100）×10^9^/L，6例患者PLT为（101～300）×10^9^/L。

3. 危险因素分析：单因素分析显示，年龄、ITP病程>1年、合并高血压、合并冠心病、合并糖尿病、PLT与发生血栓相关，差异均有统计学意义（*P*值均<0.05）。多因素分析结果表明，血栓组与无血栓组年龄、ITP病程>1年比例、合并冠心病比例、PLT的差异均有统计学意义（*P*值均<0.05），是ITP患者发生血栓的独立危险因素（表2）。

**表2:** 原发免疫性血小板减少症（ITP）患者发生血栓事件的单因素、多因素分析

因素	单因素分析		多因素分析	
	*P*值	*OR*（95％*CI*）	*P*值	*OR*（95％*CI*）
男性	0.190	1.56（0.80～3.05）		
年龄	<0.001	1.05（1.03～1.08）	0.026	1.03（1.01～1.06）
体重指数（kg/m^2^）	0.516	1.03（0.95～1.11）		
ITP病程>1年	<0.001	3.47（1.72～7.01）	<0.001	4.09（1.82～9.15）
有吸烟史	0.487	1.40（0.54～3.57）		
有饮酒史	0.262	1.93（0.61～6.09）		
合并高血压	0.002	3.01（1.51～5.99）	0.573	0.76（0.29～1.98）
合并冠心病	<0.001	11.58（5.16～26.01）	<0.001	6.13（2.22～16.95）
合并糖尿病	0.001	3.64（1.68～7.87）	0.162	2.07（0.75～5.72）
感染	0.851	1.09（0.45～2.60）		
卧床超过3 d	0.333	1.83（0.54～6.23）		
PLT（×10^9^/L）	0.002	1.01（1.01～1.02）	0.009	1.01（1.01～1.01）
LDL-C（mmol/L）	0.255	0.81（0.57～1.16）		
TC（mmol/L）	0.168	0.81（0.60～1.09）		
TG（mmol/L）	0.994	1.00（0.80～1.26）		
糖皮质激素治疗	0.925	0.97（0.49～1.92）		
IVIg治疗	0.479	0.73（0.31～1.73）		
促血小板生成药物治疗	0.937	0.97（0.47～2.00）		
利妥昔单抗治疗	0.444	1.44（0.56～3.71）		
脾切除术治疗	0.435	1.88（0.39～9.19）		
达那唑治疗	0.968	0.98（0.33～2.94）		
咖啡酸治疗	0.213	0.46（0.14～1.56）		

**注** PLT：血小板计数；LDL-C：低密度脂蛋白胆固醇；TC：总胆固醇；TG：甘油三酯；IVIg：静脉注射免疫球蛋白

4. 列线图预测模型的构建：将ITP患者发生血栓的独立危险因素（年龄、ITP病程>1年、合并冠心病、PLT）作为预测因子构建列线图预测模型（图1）。

**图1:**
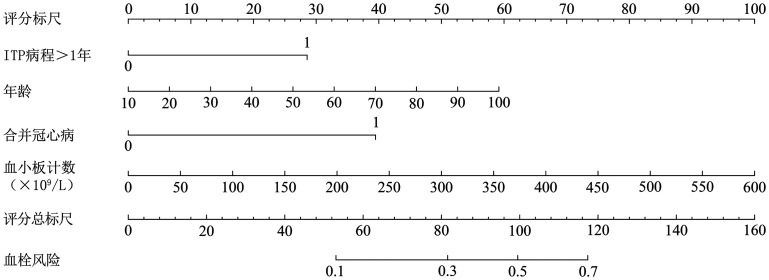
原发免疫性血小板减少症（ITP）患者发生血栓的风险预测模型 **注** 4个预测因子在图中相应坐标轴上可以找到对应点，以该点作垂直于横轴的直线，该直线与分数轴的交点为该变量的分数，各变量的分数求和得到总分，总分在血栓风险坐标轴上的读数即为ITP患者发生血栓的概率

5. 列线图预测模型的评价：列线图预测模型的受试者操作特征曲线显示，曲线下面积为0.80（95％*CI*：0.72～0.88），提示该模型区分度好（图2）。同时绘制该预测模型的校准曲线，显示校准度良好，表示预测血栓发生率与实际血栓发生率相关性良好（图3）。对风险预测模型进行Hosmer-Lemeshow拟合优度检验，结果显示，*χ*^2^＝5.838，*P*＝0.665，提示该预测模型的拟合程度较好。

**图2:**
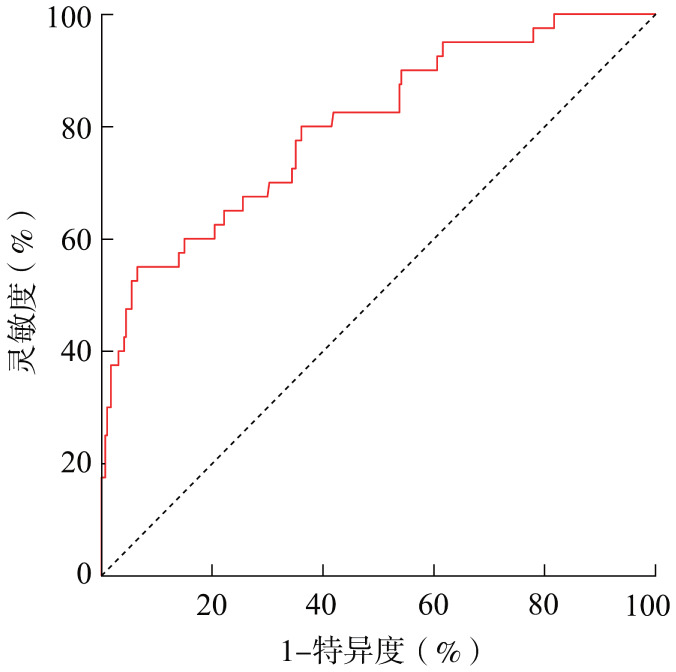
原发免疫性血小板减少症患者血栓风险预测模型的受试者操作特征曲线

**图3:**
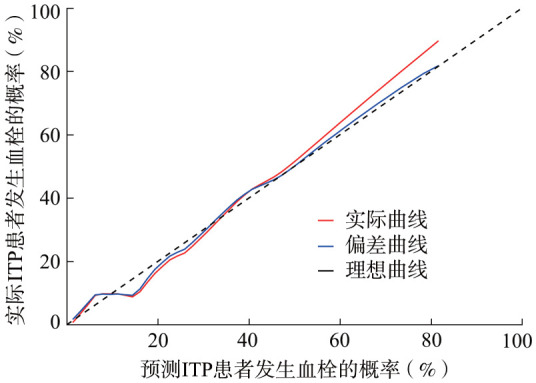
原发免疫性血小板减少症（ITP）患者血栓风险预测模型的校正曲线

## 讨论

ITP的既往研究显示，患者的出血风险随着外周血PLT的降低而增加，出血事件尤其是致命性颅内出血受到普遍重视[9]–[10]。近年来国内外的病例报道及临床研究显示，ITP患者也会发生血栓且发生率高于普通人群[11]。合并血栓会加重ITP患者的不良情绪，增加患者的经济负担，加大治疗难度。本研究积极探索ITP患者发生血栓的危险因素并构建了列线图风险预测模型，有助于临床筛选出高风险人群并对其及时干预，降低血栓发生风险。

本研究中40例（12.0％）患者发生血栓，血栓发生率高于既往文献报道[2],[12]–[13]，可能的原因是，本研究纳入的患者均为住院成人ITP患者，其中持续性与慢性ITP患者204例（61.1％），这部分患者病情反复，多次及长期住院治疗可能造成血栓发生率较高。其次，患者中位年龄偏高，75例（22.5％）合并高血压，43例（12.9％）合并糖尿病，也会导致血栓发生率升高。32例（9.58％）患者发生动脉血栓，动脉血栓的发生部位又以心、脑血管为主。静脉血栓的发生部位以外周血管更为多见。心、脑血管血栓（包括静脉血栓和动脉血栓）的发生率为84.1％，远高于其他部位。ITP患者发生血栓可能与疾病本身相关，血小板破坏增加，导致新生成的血小板增多，形成血栓的活性更强。ITP患者的血小板微粒增多，微粒表面表达的磷脂酰丝氨酸对于血栓形成至关重要。合并糖尿病、高血压、心血管疾病的ITP患者可能伴随着血管内皮的损伤，ITP相关治疗可能会快速提升血小板水平，增加血栓发生风险[14]。

本研究结果显示，年龄是ITP患者发生血栓的独立危险因素，年龄越大，ITP患者发生血栓的风险越高，与之前的研究相符[2],[12],[15]–[16]。Lafaurie等[15]基于法国健康保险系统数据库进行了针对成人ITP患者的队列研究，结果显示，随着年龄的增加，ITP患者发生动、静脉血栓的概率均增加。Ruggeri等[16]进行的意大利多中心回顾性队列研究同样发现年龄是ITP患者发生血栓的独立预测因素，年龄>60岁是重要的危险因素。在既往报道中，性别是否与ITP患者发生血栓相关仍有争议。一项基于丹麦人群的队列研究显示，慢性ITP女性患者动脉血栓的发生率是对照组的2.27倍，但男性患者动脉血栓发生率低于对照组[17]。英国GPRD研究的结果与上述研究相反，ITP男性患者动脉血栓的发生率是对照组的1.81倍，而女性患者动脉血栓发生率与对照组相比差异无统计学意义[13]。国内的回顾性研究分析了性别对ITP患者血栓发生率的影响，结果显示，男、女性患者血栓发生率的差异无统计学意义，与本研究结果一致[18]。

本研究结果显示，ITP病程>1年是ITP患者发生血栓的独立危险因素，慢性ITP病程通常持续数年，自发性缓解少见，因此随着ITP病程的延长，患者可能需要多种治疗方案，既往研究表明，多次（≥3次）治疗是血栓发生的危险因素[18]–[19]。Severinsen等[20]基于丹麦人群进行了一项队列研究，结果表明，慢性ITP患者发生静脉血栓的风险是正常人群的2倍。Nørgaard等[17],[21]的研究进一步证实，与一般人群相比，慢性ITP患者动脉血栓和静脉血栓的发生风险均增加。目前国内没有关于ITP病程与发生血栓风险相关性的研究，本研究结果显示，血栓组与非血栓组ITP病程>1年患者比例的差异有统计学意义（*P*<0.001），是ITP患者发生血栓的独立危险因素。本研究还显示，合并高血压、冠心病、糖尿病是ITP患者发生血栓的危险因素，多因素分析显示，合并冠心病是ITP患者发生血栓的独立危险因素。心血管危险因素会导致血管内皮破坏，诱发循环中血小板聚集，可能会进一步使内皮细胞激化相关抗体，或许是血栓形成的诱因。心血管危险因素还包括肥胖、吸烟史、饮酒史、血脂异常、感染等，本研究中，上述变量与血栓的发生无明显相关性（*P*值>0.05）。目前有研究认为，糖皮质激素、脾切除、促血小板生成药物可能是ITP患者发生血栓的危险因素。但也有研究认为，糖皮质激素和脾切除与发生血栓无相关性（*P*值均>0.05）[18]。Shen等[22]的研究显示，阿伐曲泊帕、艾曲泊帕、罗米司亭均未显著增加ITP患者血栓事件发生率（*P*值均>0.05）。本研究结果显示，血栓组与非血栓组糖皮质激素、IVIg、脾切除术、利妥昔单抗、促血小板生成药物、达那唑、咖啡酸等治疗的差异均无统计学意义（*P*值均>0.05）。

综上所述，当临床中遇到ITP患者合并血栓时，应及时关注，可考虑使用该列线图预测模型，评估纳入该模型的4个变量，根据血栓发生概率及时调整临床用药及治疗策略。该预测模型的建立有助于对ITP患者进行客观、有效的血栓风险评估，具有较高的准确性，可实现个体化预测并进行相应干预。但考虑到本研究纳入的是住院ITP患者，将该模型推广至门诊随访的ITP病例时，需要进一步验证其预测效能。
